# Heparanase-Neutralizing Monoclonal Antibody (mAb A54) Attenuates Tumor Growth and Metastasis

**DOI:** 10.3390/cells14171379

**Published:** 2025-09-04

**Authors:** Uri Barash, Malik Farhoud, Maali Odeh, Eliezer Huberman, Liang Wu, Israel Vlodavsky

**Affiliations:** 1 Technion Integrated Cancer Center (TICC), Rappaport Faculty of Medicine, Technion, Haifa 31096, Israel; ubarash@yahoo.com (U.B.); malik.far.96@gmail.com (M.F.); maali.odeh@gmail.com (M.O.); 2 School of Pharmacy, University of Illinois, Chicago, IL 60654, USA; elhuberman@gmail.com; 3 The Rosalind Franklin Institute, Harwell OX11 0FA, UK; liang.wu@rfi.ac.uk; 4 Division of Structural Biology, University of Oxford, Centre for Human Genetics, Oxford OX3 7BN, UK

**Keywords:** heparanase, heparan sulfate, neutralizing monoclonal antibody, tumor xenograft, tumor growth inhibition, pancreatic cancer, combination therapy

## Abstract

Heparanase is the only human enzyme responsible for heparan sulfate (HS) breakdown, an activity that remodels the extracellular matrix (ECM) and strongly drives cancer metastasis and angiogenesis. Compelling evidence implies that heparanase promotes essentially all aspects of the tumorigenic process, namely, tumor initiation, vascularization, growth, metastasis, and chemoresistance. A key mechanism by which heparanase accelerates cancer progression is by enabling the release and bioavailability of HS-bound growth factors, chemokines, and cytokines, residing in the tumor microenvironment and supporting tumor growth and metastasis. The currently available heparanase inhibitors are mostly HS/heparin-like compounds that lack specificity and exert multiple off-target side effects. To date, only four such compounds have progressed to clinical trials, and none have been approved for clinical use. We have generated and characterized an anti-heparanase monoclonal antibody (A54 mAb) that specifically inhibits heparanase enzymatic activity (ECM degradation assay) and cellular uptake. Importantly, A54 mAb attenuates xenograft tumor growth and metastasis (myeloma, glioma, pancreatic, and breast carcinomas) primarily when administered (syngeneic or immunocompromised mice) in combination with conventional anti-cancer drugs. Co-crystallization of the A54 Fab fragment and the heparanase enzyme revealed that the interaction between the two proteins takes place adjacent to the enzyme HS/heparin binding domain II (HBDII; Pro271-Ala276), likely hindering heparanase from interacting with HS substrates via steric occlusion of the active site cleft. Collectively, we have generated and characterized a novel mAb that specifically neutralizes heparanase enzymatic activity and attenuates its pro-tumorigenic effects in preclinical models, paving the way for its clinical examination against cancer, inflammation, and other diseases.

## 1. Introduction

Heparanase is a β-d-endoglucuronidase enzyme that specifically cleaves heparan sulfate (HS) chains of heparan sulfate proteoglycans (HSPGs), which are abundant components of the extracellular matrix (ECM) and cell surfaces [1,2]. Structurally, the active form of heparanase is generated from a latent 65 kDa proenzyme, which undergoes proteolytic processing into a heterodimer composed of 8 kDa and 50 kDa subunits that together form the catalytically active enzyme. The enzyme employs a conserved ‘double displacement mechanism’, involving two key catalytic amino acids—a nucleophile (Glu343) and a general acid/base proton donor (Glu225) [3,4]. The heparin/HS binding domains (HBD-I, HBD-II) are situated close to the active-site micro-pocked fold [3,4].

Heparanase activity directly and indirectly promotes several hallmarks of cancer [1,2,5]. Briefly, HS degradation weakens the ECM and basement membrane barriers, allowing cancer cells to invade surrounding tissues and enter blood/lymphatic vessels. It modulates ECM structure and recruits stromal fibroblasts, endothelial cells, and immune cells, creating a microenvironment that favors tumor growth and angiogenesis. Importantly, high heparanase expression correlates with poor prognosis, higher tumor grade, and increased metastasis in many cancers (e.g., breast, colon, lung, pancreatic, bone sarcomas, and hematologic malignancies) [1,2,5].

Heparanase is also involved in the pathogenesis of inflammation, kidney dysfunction, fibrosis, atherosclerosis, and viral infection [6,7,8,9,10], supporting the notion that heparanase is a valid drug target. A key venue by which heparanase exerts its multiple effects on cells and tissues is by regulating the bioavailability of HS-bound growth factors, chemokines, and cytokines, thereby mediating tumor-host crosstalk and tissue remodeling [2,5,11]. Among the proteins sequestered by the ECM and liberated by heparanase are typical growth-promoting and proangiogenic factors such as hepatocyte growth factor (HGF), basic fibroblast growth factor, heparin-binding epidermal growth factor, and vascular endothelial growth factor A (VEGF-A) [11,12,13].

A variety of heparanase-inhibiting molecules have been developed, including small molecules, peptides, non-anticoagulant species of heparin, and related polyanionic molecules such as M402, PI-88, SST0001 (= Roneparstat), and PG545 (= Pixatimod) [14,15,16,17,18,19,20,21]. To date, only four compounds have progressed to clinical trials [22,23,24,25], and none have been approved for clinical use. The heparin-like properties of these four ‘best-in-class’ polyanionic compounds produce undesirable off-target pleiotropic effects (e.g., anticoagulation, interaction with growth-promoting factors, poor pharmacokinetics) that hamper their clinical use. For example, heparin binds PF4 to trigger an autoimmune response, heparin-induced thrombocytopenia (HIT), regarded among the reasons why some anti-heparanase compounds were halted in clinical trials [26,27]. A newly synthesized sulfated pseudo-tetrasaccharide aminoglycoside ligand inhibits the heparanase enzyme but fails to interact with PF4, paving the way for the design of aminoglycoside-based therapeutics with minimized adverse effects associated with PF4 [26]. Given the lack of specificity and off-target effects of the current heparin/HS mimicking compounds, the present study focuses on the development of a heparanase-neutralizing monoclonal antibody (A54 mAb). We characterized the interaction of this mAb with the heparanase protein and demonstrated its efficacy in preclinical models of cancer, administered alone and in combination with conventional anti-cancer drugs.

## 2. Materials and Methods

### 2.1. Generation of Monoclonal Antibodies (mAb)

BALB/c mice were immunized with 50 µg of recombinant 65 kDa latent heparanase in complete Freund’s adjuvant (CFA; Sigma, St. Louis, MO, USA), followed by five injections (50 µg) of heparanase in incomplete Freund’s adjuvant (IFA) every 2 weeks. Splenocytes were then isolated, fused with NSO myeloma cells, and the resulting hybridomas were screened for their ability to bind heparanase by ELISA, essentially as described [28,29,30]. Positive hybridomas were selected, cloned, and examined for inhibition of heparanase enzymatic activity utilizing sulfate-labeled, naturally deposited ECM as substrate [31,32]. Hybridoma A54, found to neutralize the enzymatic activity of heparanase most effectively, was selected for subsequent experiments. The hybridoma subclass was determined by isotyping kit according to the manufacturer’s (Abcam, Cambridge, UK) instructions. mAb A54 was characterized as IgG1 and was purified by affinity chromatography on Protein G Sepharose-4 according to the manufacturer’s (Cytiva, GE Healthcare, Marlborough, MA, USA) instructions. While the purified A54 mAb was applied in our initial experiments, we have subsequently generated a recombinant version of the mAb for the sake of reliability, batch consistency, scalability, and potential modifiability. All steps (RNA isolation, cDNA synthesis, VH and VL gene amplification, cloning into pcDNA3.4 vector, expression in HEK293/Expi293F cells, purification, and validation (SDS-PAGE, Western blotting) were performed by GenScript Biotech (Singapore, 349248), including the determination of the CDRs of the recombinant mAb. Notably, the recombinant A54 mAb was applied in all the in vivo studies reported in the present study.

### 2.2. Cells and Cell Culture

Human HEK 293 and U87-MG glioma and mouse EMT-6 breast carcinoma cells were purchased from the American Type Culture Collection (ATCC, Manassas, VA, USA). MPC-11 [28] and CAG [33] myeloma (kindly provided by Dr. RD Sanderson, UAB), U87 glioma [34], and EMT-6 breast carcinoma [35] cells have been described previously. KPC cells were kindly provided by Dr. E. Hasnis (Rappaport Faculty of Medicine, Technion). Cell lines were confirmed to be pathogen-free, and human cell lines were authenticated to verify their origin before use. Cells were grown in Dulbecco’s modified Eagle’s medium (Biological Industries, Beit Haemek, Israel) supplemented with 10% fetal calf serum and antibiotics.

### 2.3. Heparanase Enzymatic Activity—ECM Degradation Assay

Sulfate [^35^S] labeled ECM, prepared as described [31], was incubated with recombinant human heparanase in the absence and presence of the A54 mAb. To evaluate the release of HS degradation fragments, the incubation medium was collected and applied for gel filtration onto Sepharose 6B. Fractions (0.2 mL) were eluted with PBS and counted for radioactivity. Degradation fragments of HS side chains were eluted from Sepharose 6B at 0.5 < Kav < 0.8 (peak II) [30,32]. For inhibition studies, recombinant heparanase (200 ng) was preincubated with the antibody for 30 min on ice at pH 7.2 before being incubated at pH 5.8 with ^35^S-labeled ECM, used as a substrate for the heparanase enzyme [31,32]. For more information, see Supplementary Materials.

### 2.4. Matrigel Invasion Assay

Cell invasion through Matrigel was performed using modified Boyden chambers with polycarbonate Nucleopore membrane, essentially as described [36,37]. For more information, see Supplementary Materials.

### 2.5. Tumor Models

Mice were housed in a pathogen-free facility with access to food and water ad libitum. All animal studies were performed in compliance with the regulations and ethical guidelines for experimental animal studies, in accordance with the Technion’s Institutional Animal Care and Use Committee (IL-165-12-2024; OPRR-A5027-01).

### 2.6. U87 Glioma

Luciferase-labeled U87 glioma cell suspension (5 × 10^6^/0.1 mL) was inoculated subcutaneously (s.c.) at the right flank of 5-week-old female NOD/SCID mice. Three days after cell inoculation, mice are randomly assigned to two groups (5 mice each), receiving either vehicle (PBS) or A54 mAb (500 µg/mouse, i.p., three times/week). Tumor development was inspected (once a week) by IVIS imaging. For more information, see Supplementary Materials.

### 2.7. CAG Myeloma

Luciferase-labeled CAG human myeloma cells (5 × 10^6^) are injected into the tail vein of NOD/SCID mice. Three days after cell inoculation, mice are randomly assigned to 2 cohorts (5 mice each) receiving (a) vehicle (PBS), and (b) A54 mAb (500 µg/mouse, 3 times/week). Tumor development is monitored (once a week) using IVIS imaging following the administration of luciferin [30,33].

### 2.8. MPC-11 Myeloma

Mouse MPC-11 myeloma/plasmacytoma cells were detached, washed with PBS, brought to a concentration of 5 × 10^5^ cells/0.2 mL, and inoculated subcutaneously at the right flank of 6-8-week-old Balb/c mice. Three days after cell inoculation, mice are randomly assigned to 2 cohorts (6 mice each) receiving (a) vehicle (PBS), and (b) A54 mAb (250 or 500 µg/mouse, 3 times/week). Xenograft size is determined by externally measuring tumors in 2 dimensions. At the end of the experiment, mice are sacrificed, and tumor xenografts are removed and weighed [28].

### 2.9. Pancreatic Cancer

C57BL/6 mice were inoculated (s.c.) with mouse Panc02 or KPC cells (1 × 10^6^/mouse), and mice were treated with A54 mAb (i.p., 360 µg/mouse, 2 times/week), Gemcitabine (Gem; 30 mg/kg twice a week), A54 mAb + Gemcitabine (com) or vehicle alone (PBS) as control. Tumor development was calculated from external caliper tumor measurements. At the end of the experiment, tumors were resected, photographed, and weighed.

### 2.10. Crysalization of the Enzyme–Antibody Complex

Mature heparanase protein was produced and purified as previously described (3, 4). To investigate the interaction between the A54 mAb and heparanase, Fab fragments were prepared from purified A54 mAb via ficin protease digest using the mouse IgG1 Fab and F(ab’)2 preparation kit (Thermo Fisher Scientific, Waltham, MA, USA) following the manufacturer’s protocols. The isolated Fab fragments were further purified by size-exclusion chromatography using a Superdex S200 16/600 column, and the buffer was exchanged into 20 mM HEPES (pH 7.4), 200 mM NaCl, and 1 mM DTT. Purified FabA54 was mixed with purified heparanase at a ~2:1 Fab: Heparanase ratio, incubated at room temperature for 2 h, and purified again by size exclusion chromatography to remove unbound Fab fragments. The purified FabA54–heparanase complex was then concentrated to 5.4 mg/mL and subjected to crystallization using commercially available screens. Crystals were found in the PACT Premier crystallization screen (Molecular Dimensions Ltd., Calibre Scientific UK, Sheffield, UK), condition E5 (0.2 M sodium nitrate, 20% polyethylene glycol 3350). Crystals were transferred to a cryoprotectant solution (0.2 M sodium nitrate, 20% polyethylene glycol 3350, and 25% ethylene glycol) before harvesting and flash-cooling in liquid nitrogen for X-ray data collection. X-ray diffraction data were collected on beamline I04-1 of the Diamond Light Source, Didcot, UK, and processed using the XDS and STARANISO pipelines [38,39]. The dataset was phased by molecular replacement using the structures of unliganded heparanase (PDB accession code 5E8M) and an unrelated mouse IgG Fab fragment (PDB accession code 1AE6). The structure was further improved by iterative rounds of manual model building and maximum-likelihood refinement using COOT and REFMAC5, respectively [40,41]. Refinement was carried out using TLS restraints, jelly body restraints, and Prosmart restraints [42] using 5E8M and 1AE6 as reference models. Binding analyses were carried out on the solved crystal structure using PISA [43]. The HPSE-A54 structure has been deposited in the PDB under accession code 9S8W.

### 2.11. Statistics

Each experiment was performed 2–3 times, and the variation between individual experiments did not exceed ± 15% of the mean. Representative experiments are presented in the figures. Data are presented as mean ± SEM. Statistical significance was analyzed by the two-tailed Student’s *t*-test or one-way ANOVA. The value of *p* < 0.05 was considered significant.

## 3. Results

### 3.1. Generation and Characterization of Heparanase-Neutralizing mAb

**Figure 1:**
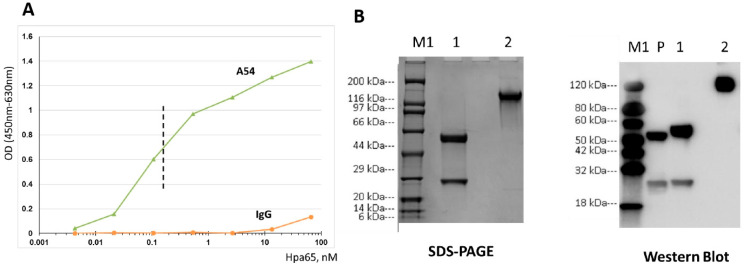
Characterization of neutralizing A54 anti-heparanase monoclonal antibody. (**A**). A54 mAb preferentially recognizes heparanase. Microtiter plate was coated with Latent 65 kDa heparanase (Hpa65). A54 mAb and control mouse IgG were added at the indicated concentrations, and ELISA was used to determine binding to the immobilized enzyme. (**B**). SDS-PAGE (left) and Western blot (right) analyses of A54 mAb heavy and light chains. Lanes M1: Protein markers Lane 1: Reducing condition Lane 2: Non-reducing condition Lane P: Mouse IgG1-Kappa as a positive control. (Secondary WB antibody = Goat Anti-Mouse IgG-HRP).

To select the hybridoma clone that best inhibits heparanase enzymatic activity, supernatants of the various hybridomas, raised against the 65 kDa latent heparanase protein, were collected, pre-incubated with recombinant heparanase, and examined for the capacity to inhibit the production and release of heparan sulfate (HS) degradation fragments from sulfate-labeled ECM [30,32,34]. For this purpose, purified recombinant active heparanase was pre-incubated with the various hybridoma supernatants for 2 h in serum-free RPMI medium on ice. The mixture was then applied onto ^35^S-labeled ECM-coated dishes, and heparanase activity was determined as described under “Materials and Methods”. Hybridoma #A54 yielded the best heparanase-inhibiting activity (nearly 80% decrease in the amount of released sulfate-labeled HS degradation fragments) compared to the other 24 hybridoma supernatants, which yielded little (up to 30%) or no heparanase-inhibiting effect (not shown). A54 mAb was purified (protein G Sepharose) from the hybridoma and subjected to ELISA to validate the preferential recognition of heparanase by A54 mAb vs. control mouse IgG (Figure 1A). Briefly, a microtiter 96-well plate was coated with latent 65 kDa heparanase (Hpa65). Purified mAb A54 or control mouse IgG were then added at the indicated concentrations, and their binding to the immobilized heparanase was determined. While there was no interaction of heparanase with control mouse IgG, the A54 mAb exhibited a high affinity binding to the enzyme (IC50 = 0.2 nM, Kd = 3.18 × 10^−9^ M determined by Biacore analysis). Given the low yield of purified A54 mAb and the large amounts needed for the in vivo experiments, we decided to produce and use recombinant mAb in all the subsequent experiments. All steps involved in cloning, production, and characterization of the recombinant A54 mAb were performed by GenScript Biotech (Singapore). The recombinant A54 mAb was subjected to SDS-PAGE and Western blot analyses and characterized as IgG1-Kappa (Figure 1B).

### 3.2. A54 mAb Inhibits Heparanase Enzymatic Activity, Cellular Uptake, and Cell Invasion

#### 3.2.1. Enzymatic Activity

**Figure 2:**
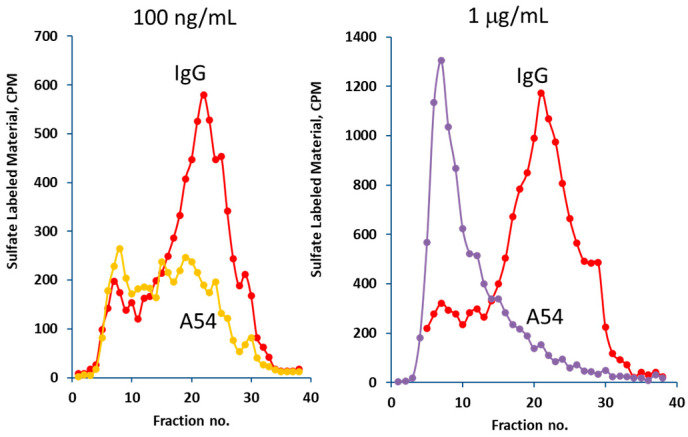
A54 mAb inhibits heparanase enzymatic activity. Purified recombinant active heparanase (200 ng) was pre-incubated with control mouse IgG or A54 mAb at 0.1 μg/mL (**left**) or 1 μg/mL (**right**) for 1 h in serum-free RPMI medium on ice. The mixture was then applied onto dishes coated with ^35^S-labeled ECM, and heparanase enzymatic activity (release of sulfate-labeled fragments of HS into the incubation medium) was determined as described in ‘Methods’.

The ability of the purified recombinant mAb A54 to inhibit heparanase enzymatic activity was examined. For this purpose, recombinant active heparanase was preincubated with control mouse IgG or mAb A54, followed by determination of heparanase enzymatic activity. As shown in Figure 2 (left), 0.1 µg/mL of the antibody yielded nearly 75% inhibition of the enzyme. Complete inhibition (e.g., release from the ECM of sulfate-labeled material subjected to gel filtration on Sepharose 6B and eluted in fractions # 15–30) was obtained in the presence of 1 µg/mL of the antibody (Figure 2, right). There was no inhibition by the control mouse IgG.

#### 3.2.2. Heparanase Uptake and Processing

**Figure 3:**
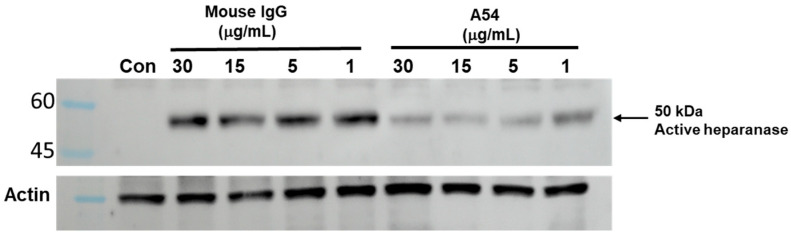
A54 mAb inhibits cellular uptake and processing of exogenously added heparanase. HEK 293 cells were incubated (overnight) with latent 65 kDa heparanase (1 µg/mL) in the absence (Con) or presence of increasing concentrations (1, 5, 15, 30 µg/L) of A54 mAb or control mouse IgG. The cell medium (serum-free) was then aspirated, and cell lysates were subjected to immunoblotting, applying anti-heparanase (upper panel) or anti-actin (lower panel) polyclonal antibodies [45,46].

Heparanase is readily secreted but does not normally accumulate extracellularly due to rapid interaction with HS on the cell surface, followed by internalization and processing into the active enzyme [44,45]. Heparanase uptake is a prerequisite for delivery of the latent enzyme to lysosomes and its subsequent proteolytic processing and activation [44,45]. To investigate the effect of the A54 mAb on this process, HEK293 cells were incubated overnight with latent 65 kDa heparanase in the absence or presence of increasing concentrations of the A54 mAb or control mouse IgG1. The cell medium (serum-free) was aspirated, and cell lysates were subjected to immunoblotting, applying polyclonal anti-heparanase (upper panel) or anti-actin (lower panel) antibodies [45]. As demonstrated in Figure 3, inhibition of heparanase uptake and activation was obtained already in the presence of 1 µg/mL A54 mAb (molar ratio of 2.3:1 (Hpa:A54). There was no inhibition by non-immune mouse IgG. This result indicates that A54 mAb not only inhibits heparanase enzymatic activity but also attenuates the uptake and processing of the latent enzyme, thus providing an additional mechanistic aspect that contributes to its potent anti-tumorigenic activity.

#### 3.2.3. Cell Invasion

**Figure 4:**
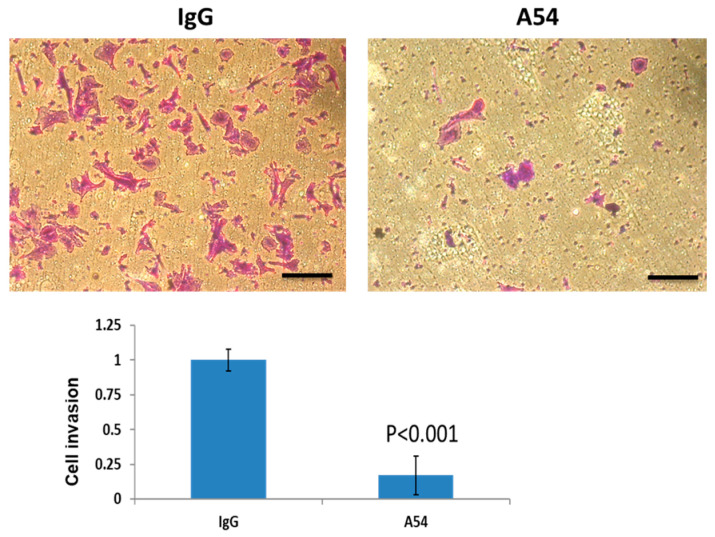
A54 mAb attenuates cell invasion. U87 glioma cells (1 × 10^5^) were plated onto Matrigel-coated 8-μm trans-well filters in the presence of control mouse IgG (**left**) or A54 mAb (2 μg/mL) (**right**). Six hours after seeding, invading cells adhering to the lower side of the membrane were stained (crystal violet, upper panels) and quantified (IgG set arbitrarily to 1; lower panel). Scale bar = 100 microns.

We examined the effect of the A54 mAb on cell invasion. Briefly, U87 glioma cells were plated onto Matrigel-coated transwell filters in the presence of control mouse IgG or mAb A54. Invading cells adhering to the lower side of the membrane were visualized after 6 h (Figure 4, top) and quantified (Figure 4, bottom). As presented in Figure 4 invasion of U87 glioma cells through Matrigel (reconstituted basement membrane) was attenuated by nearly 85% in the presence of A54 mAb.

#### 3.2.4. Anti-Heparanase A54 mAb Attenuates Myeloma and Glioma Tumor Growth

**Figure 5:**
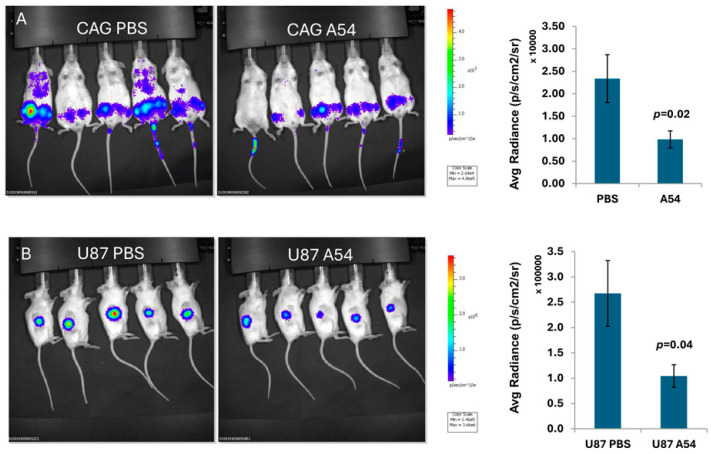
A54 mAb attenuates tumor growth. (**A**) CAG human myeloma. NOD/SCID mice (n = 5) were inoculated (i.v.) with CAG-luciferase cells (5 × 10^6^), and the mice treated with A54 mAb (500 µg/mouse, 3 times/week) or PBS as control. (**B**) U87 human glioma. NOD/SCID mice (n = 5) were inoculated (s.c.) with U87-luciferase cells (5 × 10^6^), and mice were treated with A54 mAb (i.p., 500 µg/mouse, 3 times/week) or PBS as a control. Tumor growth was evaluated by IVIS imaging on day 20 (left). Quantification of the luciferase signals is shown graphically (right).

**Figure 6:**
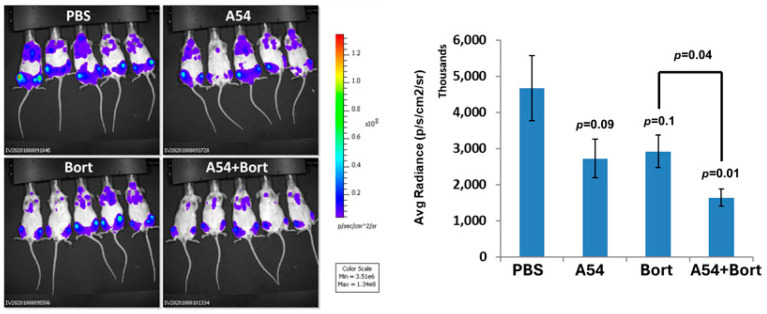
Combination of A54 mAb and bortezomib attenuates myeloma tumor growth. CAG myeloma. NOD/SCID mice (n = 5) were inoculated (i.v.) with CAG-luciferase cells (5 × 10^6^), and the mice treated with vehicle alone (PBS), A54 mAb (i.p., 360 µg/mouse, 2 times/week), Bortezomib (Bort; 0.5 mg/kg twice a week), or both (A54 mAb + Brotezomib). Tumor growth was evaluated by IVIS imaging (**Left**, day 23); quantification of the luciferase signals is shown graphically (**Right**).

The ability of A54 mAb to attenuate myeloma tumor development over time was examined. Briefly, NOD/SCID mice were inoculated intravenously (i.v.) with Luc-CAG human myeloma cells. The mice were treated with A54 mAb (500 µg/mouse, 3 times/week) or PBS as a control, starting on day 3 following cell inoculation, and tumor growth was evaluated once a week by IVIS imaging (Figure 5A). Quantification of the luciferase signals on day 22 is shown graphically (Figure 5A, upper right), indicating a marked 66% reduction in myeloma tumor cell dissemination, colonization, and growth (*p* = 0.02). Given that the A54 mAb was administered 3 days after intravenous inoculation of the CAG cells, it is likely that cell invasion was already completed and that the IVIS imaging reflects cell colonization and growth in bone. Next, Luc-U87 human glioma cells were inoculated subcutaneously (s.c.) at the right flank of 5-week-old female NOD/SCID mice. Mice were treated with mAb A54 or PBS as described above. Tumor growth was evaluated by IVIS imaging (Figure 5B), and quantification of the luciferase signals is shown graphically (Figure 5, lower right), revealing ~70% reduction in glioma tumor growth in the A54-treated mice (*p* = 0.04). In subsequent studies, we investigated the effect of mAb A54 administered in combination with bortezomib on myeloma tumor growth. Briefly, NOD/SCID mice were inoculated (i.v.) with CAG luciferase cells, and mice were treated with A54 mAb (360 µg/mouse, 2 times/week), Bortezomib (Bort; 0.5 mg/kg twice a week), or with a combined A54 mAb + Bortezomib protocol. Tumor growth was evaluated by IVIS imaging (Figure 6, left panels), and quantification of the luciferase signals is shown graphically (Figure 6, Right). The results indicate that the A54 mAb is effective as monotherapy and that the combined treatment was more effective than each drug alone.

**Figure 7:**
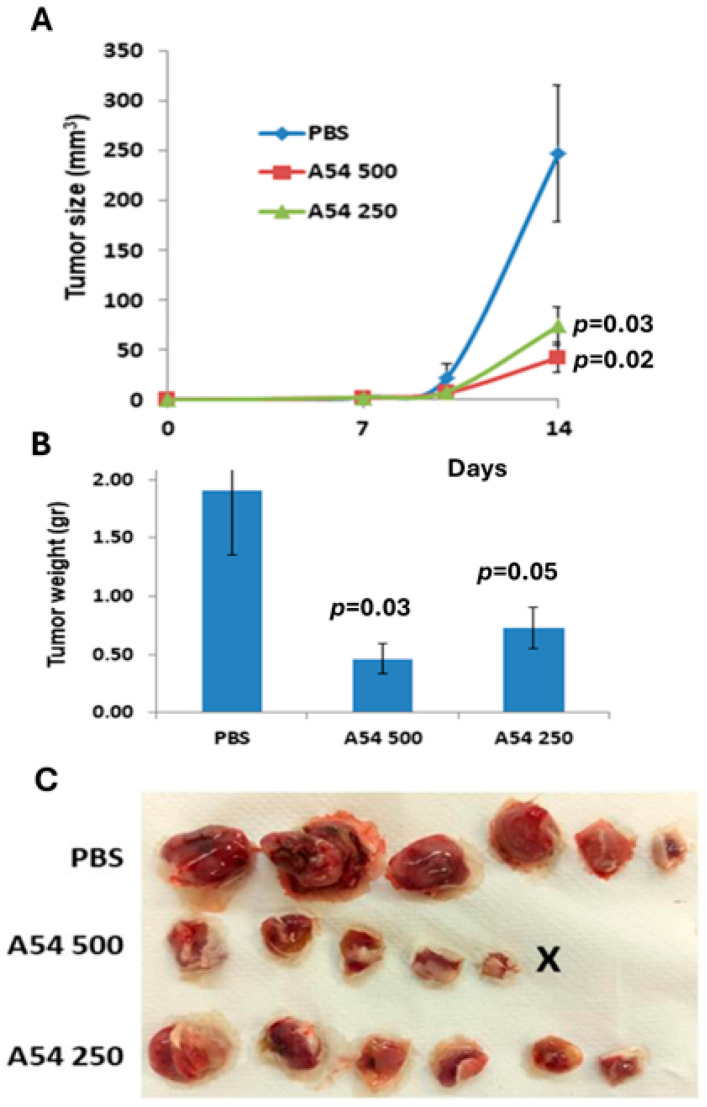
A54 mAb attenuates mouse myeloma/plasmacytoma tumor growth. Line graph (**A**), bar graphs (**B**), and photographs (**C**) representing the effect of mAb A54 (i.p., 250 and 500 µg/mouse, i.p., twice a week) in attenuating MPC-11 mouse plasmacytoma tumor growth in syngeneic Balb/c mice, compared to tumor-bearing mice treated with PBS (control). At the end of the experiment (day 14), mice were sacrificed, and the tumor xenografts were removed and weighed. X = Tumor could not be detected.

Having demonstrated the inhibitory effect of A54 mAb in immunocompromised mouse models, we subsequently examined the effect of the antibody in a syngeneic (immunocompetent) mouse model. Briefly, Balb/c mice were inoculated (s.c.) with MPC-11 mouse myeloma/plasmacytoma cells. Treatment with A54 mAb (250 or 500 µg/mouse, twice a week) was initiated two days after cell inoculation, while control mice were administered PBS alone. Xenograft size was determined twice a week by externally measuring the tumors in two dimensions, and tumor volume was calculated (Figure 7A). At the end of the experiment (Day 14), xenografts were removed, weighed (Figure 7B), and photographed (Figure 7C). The results indicate that treatment with mAb A54 markedly attenuated (4–5 fold) the growth of syngeneic primary myeloma tumors.

The A54 mAb was also effective in attenuating the dissemination of EMT6 breast carcinoma cells in a spontaneous metastasis model. Briefly, luciferase-labeled EMT6 breast carcinoma cells were injected into the third mammary fat pad of Balb/c mice, and treatment with mAb A54 began 3 days after cell inoculation. On day 15, the mammary fat pad, including the primary tumor, was excised. The mice were further treated with mAb A54 and subjected to IVIS bioluminescent imaging on day 20 after mastectomy. Our preliminary results (Supplementary Figure S1) indicate a marked reduction in cell dissemination in the A54-treated mice.

#### 3.2.5. Combination of A54 mAb and Gemcitabine Attenuates Pancreatic Tumor Growth

**Figure 8:**
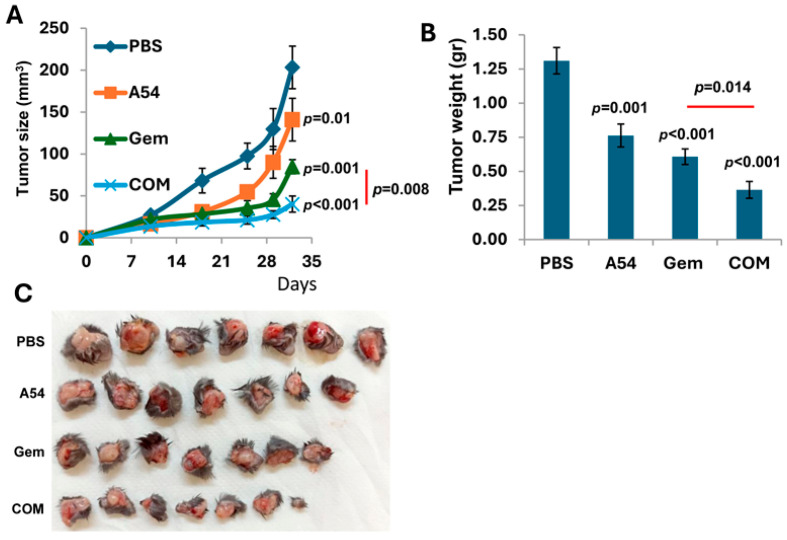
Combined treatment with A54 mAb and gemcitabine attenuates Panc02 mouse PDAC. C57BL/6 mice (n = 7) were inoculated (s.c.) with Panc02 cells (1 × 10^6^/mouse), and mice were treated with A54 mAb (i.p., 360 µg/mouse, 2 times/week), Gemcitabine (Gem; 30 mg/kg twice a week), A54 mAb + Gemcitabine (com) or vehicle alone (PBS) as control. Tumor development was calculated from external caliper tumor measurements (**A**). At the end of the experiment on day 32, tumors were resected, weighed (**B**), and photographed (**C**).

**Figure 9:**
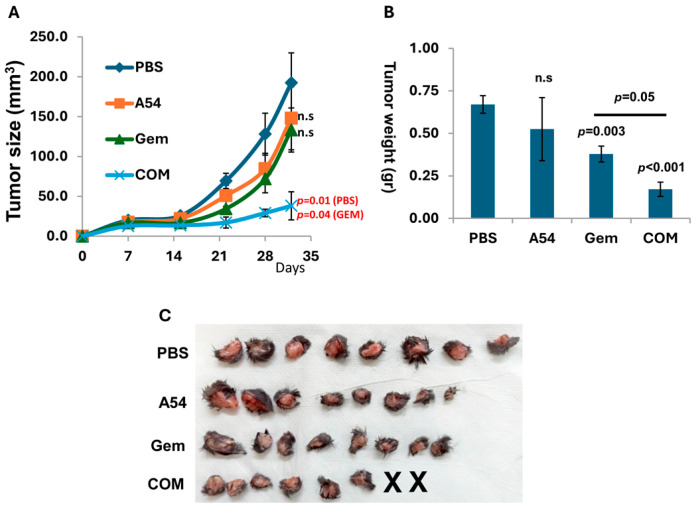
Combination of A54 mAb and gemcitabine attenuates KPC pancreatic tumor growth. C57BL/6 mice (n = 8) were inoculated (s.c.) with KPC cells (1 × 10^6^/mouse), and mice were treated with A54 mAb (i.p., 360 µg/mouse, 2 times/week), Gemcitabine (Gem; 30 mg/kg twice a week), A54 mAb + Gemcitabine (com), or vehicle alone (PBS) as control. Tumor development was calculated from external caliper tumor measurements (**A**). At the end of the experiment on day 32, tumors were resected, weighed (**B**), and photographed (**C**). X, X = Tumors could not be detected.

We investigated the effect of A54 mAb on pancreatic tumor growth. Briefly, C57BL/6 mice were inoculated (s.c.) with mouse Panc02 cells, and the mice were treated with A54 mAb (360 µg/mouse, 2 times/week), Gemcitabine (Gem; 30 mg/kg twice a week), A54 mAb + Gemcitabine, or vehicle (PBS) alone. Tumor development was calculated from external caliper tumor measurements (Figure 8A). At the end of the experiment on day 32, tumors were resected, weighed (Figure 8B) and photographed (Figure 8C). The results indicate that the combined treatment was more effective than gemcitabine alone (*p* < 0.014). Similar results were obtained with KPC mouse PDAC cells. While each drug alone yielded a small effect, the combined (A54 + gemcitabine) treatment yielded a highly significant synergistic inhibition (*p* < 0.005) of tumor growth (Figure 9).

#### 3.2.6. Humanized A54 Antibody

To reduce the immunogenicity of the A54 antibody once administered to humans, the mouse antibody was subjected to humanization (service performed by GenScript Inc., Singapore). Briefly, the Complementarity Determining Regions (CDRs) from the mouse antibody were grafted onto the human antibody frameworks that provide structural support for the variable regions. The humanized antibody was expressed in 293 HEK (Expi293F) cells and tested for binding affinity, specificity, and biological activity. While there was a 3-fold decrease in the binding affinity between the heparanase protein and the humanized vs. the mouse antibody (~3 nM vs. 1 nM), both preparations inhibited the enzyme to a comparable extent, yielding complete inhibition at 1 μg/mL (Supplementary Figure S2). Next, we examined the ability of the humanized A54 antibody to inhibit the growth of Panc02 tumors in syngeneic C57BL/6 mice. Briefly, mice were injected (s.c.) with 1 × 10^6^ Panc-02 cells in 0.1 mL PBS. The humanized A54 antibody (0.643 mg/mouse) was administered (i.p.) 3 times a week, and the tumor volume was measured on days 13, 17, and 22. The mice were sacrificed on day 26, and the tumors resected and weighed. As demonstrated in Supplementary Figure S3, tumor volume and weight were significantly inhibited (*p* = 0.0192) in mice treated with the humanized antibody.

#### 3.2.7. Structural Features of the Interaction Between A54 and Heparanase Unravel HBD-II as the Predominant Epitope

**Figure 10:**
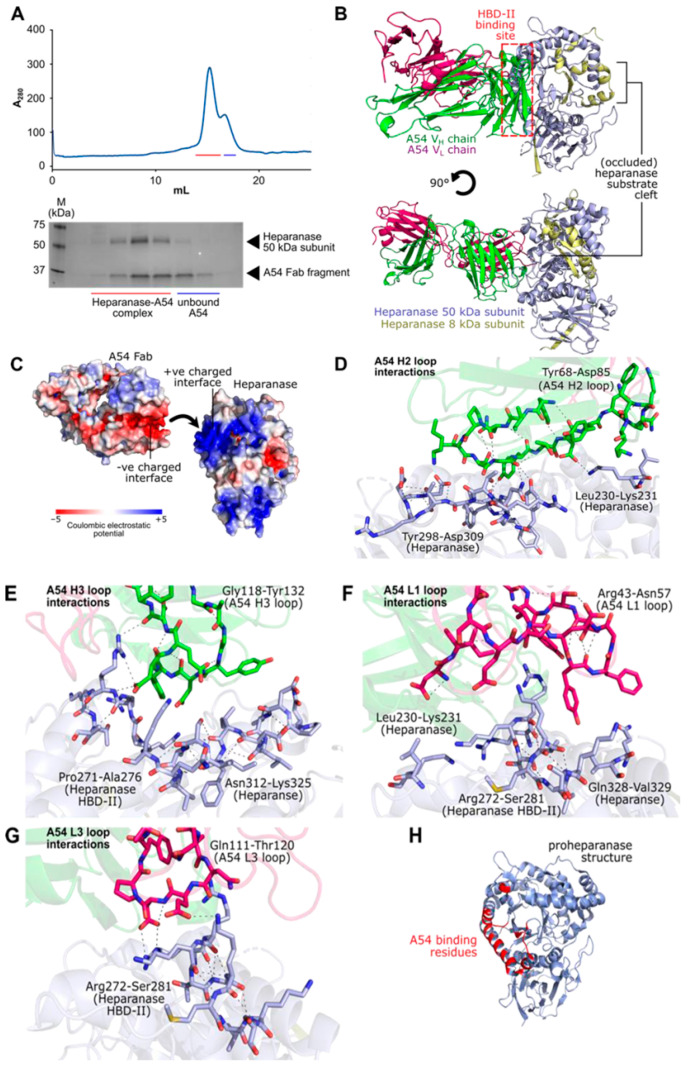
Co-crystallization of A54 and heparanase. (**A**) SEC purification and SDS-PAGE analysis of the heparanase-A54 Fab complex. (**B**) 3-dimensional structure of heparanase-A54 mAb complex, showing binding around the enzyme HBD-II region. Heparanase subunits are colored yellow (8 kDa) and purple (50 kDa). A54 mAb Fab light and heavy chains are colored in pink and green, respectively. (**C**) Surface charge analysis of A54 mAb and heparanase, highlighting the electrostatic nature of the binding interaction. HBD-II is substantially positively charged (Blue), whereas the binding interface of mAb A54 is negatively charged (Red). (**D**–**G**) Close-up views of the interactions between A54 CDR loops and heparanase amino acid residues. (**H**) Structure of pro-heparanase, with A54 mAb binding residues highlighted (based on PISA analysis; Supplementary Tables S1 and S2), illustrating the feasibility of antibody binding to the proenzyme.

To directly characterize the mode of interaction between heparanase and the A54 mAb, we further sought to solve the structure of the enzyme-antibody complex using X-ray crystallography. For this purpose, Fab fragments were prepared from purified A54 mAb via ficin protease digest, followed by reconstitution and purification of the bound complex using size-exclusion chromatography (Figure 10A). Purified heparanase-Fab_A54_ was tested against a panel of crystallization screens, leading to well-diffracting crystals in the presence of 0.2 M sodium nitrate, 20% polyethylene glycol 3350. The resulting heparanase-FabA54 X-ray diffraction dataset was processed to a resolution of 3.54 Å and was solved by molecular replacement using unliganded heparanase (PDB 5E8M) and a generic mouse IgG Fab (PDB 1AE6) as search models (Supplementary Table S1; Supplementary Figure S4).

The crystal structure of heparanase-Fab_A54_ (PDB accession code 9S8W) contains a single copy of the bound complex in each asymmetric unit. As expected, Fab_A54_ was primarily found to engage heparanase via its CDR loops, which bound heparanase across a broad binding interface that included the known HS-interacting HBD-II domain (residues Pro271-Ala276; Figure 10B). A54 binding interactions were primarily electrostatic in nature, occurring between positively charged residues on heparanase, and a cluster of negatively charged Fab CDR residues (Figure 10C). We also identified salt-bridges between Glu78_A54-VH_-Lys231_HPSE_, Lys93_A54-VH_-Asp309_HPSE_, Asp123_A54-VH_-Lys325_HPSE_, Asp125_A54-VH_-Arg273_HPSE_, and Glu116_A54-VL_-Lys277_HPSE_. Additional cation-π interactions were also noted between Tyr55_A54-VL_-Arg273_HPSE_, and H-bonds between Tyr76_A54-VH_-Tyr299_HPSE_ (Figure 10D–G). Full PISA analysis of the A54-heparanase binding interface is summarized in Supplementary Tables S2 and S3.

The complex of heparanase-A54 mAb reveals a steric hindrance mechanism for inhibition of enzyme activity by A54, as A54 binding did not perturb the position of the essential catalytic residues (nucleophile Glu343; acid/base Glu225) within the heparanase active site (Supplementary Figure S5). Instead, the presence of a bulky antibody directly around the HBD-II region of heparanase likely blocks access for long HS substrates to the enzyme substrate cleft, with neutralization of positive charge around this cleft by A54 mAb, thereby further weakening HS substrate affinity. Notably, the binding epitope for A54 mAb is retained between the proenzyme and mature forms of heparanase, rationalizing the ability of the antibody to bind to pro-heparanase to inhibit its cellular uptake and maturation (Figure 10H).

## 4. Discussion

Compelling evidence indicates that heparanase is a viable target for therapy in cancer, inflammation, autoimmunity, fibrosis, diabetes, kidney dysfunction, viral infections, and other diseases, a view widely accepted by researchers in both academia and pharma, and the topic of a dedicated book and multiple reviews [1,2,5,6,7,8,9,10,14,15]. Despite years of research, anti-heparanase-based therapy has not yet been approved for implementation in the clinic. Notably, the four heparanase-inhibiting compounds that were examined in clinical trials [22,23,24,25] are all heparin/HS mimetics that competitively target the HS binding site of heparanase by preventing HS from entering the catalytic structural domain. Although these compounds were successful in mouse models [18,19,20], adverse effects were noted in clinical studies, attributed primarily to poor pharmacokinetics, heterogeneous nature, and pleiotropic off-target effects of the lead HS mimetics [23,24,25,26]. Second-generation heparanase inhibitors, for example, in the form of disaccharides that covalently bind to the enzyme active site [3,4], appear to be devoid of such side effects and may also target cytoplasmic and nuclear activities of heparanase, thus better neutralizing all aspects of its functions.

Monoclonal antibodies (i.e., Herceptin, Rituximab, Cetuximab, Bevacizumab, Nivolumab, Pembrolizumab, Cemiplimab, etc.) revolutionized cancer treatment, offering new avenues for targeting cancer cells while minimizing damage to healthy tissue [47]. Anti-heparanase polyclonal antibodies were found to neutralize heparanase enzymatic activity and inhibit cell invasion [44], proteinuria [48], and neointima formation [49]. Moreover, neutralizing anti-heparanase monoclonal antibodies were reported to attenuate lymphoma and myeloma tumor growth in mouse models [30], but were never examined in patients. In a previous study, we have focused on the Lys158-Asp171 domain (= HBD-I) of heparanase since the respective peptide (termed KKDC) physically interacts with heparin and HS with high affinity and inhibits the heparanase enzyme [46]. mAb 9E8, directed against the KKDC peptide, was found to inhibit heparanase enzymatic activity, cell invasion, and tumor metastasis [30], the hallmark of heparanase function. mAb 9E8 also decreased the uptake of latent and active heparanase [30], an HS-dependent cellular mechanism thought to limit extracellular retention of the enzyme [45]. Not surprisingly, the 9E8 mAb has no cytotoxic effect on the tumor cells, suggesting that it affects the tumor microenvironment. This was best demonstrated with Raji lymphoma cells that lack intrinsic heparanase activity. We concluded that the ability of mAb 9E8 to inhibit the growth of Raji tumors (30) is due to the neutralization of heparanase contributed by cells (i.e., macrophages) residing in the tumor microenvironment [30]. Being an IgM mAb, the clinical significance of 9E8 mAb is limited due to production challenges, short half-life, limited tissue penetration, aggregation, and immunogenicity [30].

The A54 mAb (IgG1 subclass), raised against the full-length latent heparanase was found to inhibit heparanase enzymatic activity (ECM degradation assay), cellular uptake and cell invasion (Matrigel assay). Importantly, the antibody attenuated tumor growth and metastasis (myeloma, glioma, pancreatic cancer, breast carcinoma, all expressing heparanase enzymatic activity) in both immunocompetent (syngeneic) and immunocompromised mouse models. Best results were obtained when the mAb was administered in combination with conventional anti-cancer drugs. Notably, A54 mAb not only inhibits heparanase enzymatic activity but also attenuates the uptake and activation of the latent enzyme, providing an additional mechanistic aspect for its anti-tumorigenic mode of action. Heparanase is a multifaceted protein having both enzymatic and non-enzymatic activities. Given that some variants of the heparanase protein (i.e., C-terminus domain, active site double-mutant, T5 splice variant) that lack enzymatic activity still promote tumor progression [50,51,52], a common concern is whether the enzymatic activity of heparanase is indeed the most critical determinant of its pro-tumorigenic and pro-metastatic effects. Ongoing studies are aimed at elucidating whether the A54 mAb also inhibits some of the non-enzymatic activities of heparanase, such as signal transduction and gene transcription.

To identify the epitope and decipher the mechanism of action of mAb A54, we co-crystallized its Fab fraction with the heparanase protein. It was found that A54 Fab interacts with the (β/α)_8_-barrel domain of heparanase, right above HBD-II (heparin/HS binding domain II; Pro271-Ala276), suggesting that mAb A54 is directed against the HBD-II epitope. The binding interactions were primarily electrostatic, between the positively charged residues within HBD-II and a cluster of negatively charged Fab CDR residues. This may halt heparanase from interacting with HS substrates via steric occlusion of the active site cleft, preventing the interaction of HS chains with the heparanase active site residues. Occlusion of HBD-II by the A54 mAb may also halt the interaction of HS with this domain in a charge-based manner. Our results highlight the critical role of HBD-II in enabling a proper interaction of HS with the enzyme active site (Glu225, Glu343), resulting in endoglucuronic cleavage of the HS substrate. This result was somewhat unexpected given the previously emphasized importance ascribed to HBD-I [49]. It appears that blocking either HBD-I or HBD-II can halt the accessibility of HS to the enzyme active site and hence inhibit its cleavage. Notably, A54 mAb binding did not perturb the position of the essential catalytic residues (nucleophile Glu343; acid/base Glu225) within the heparanase active site (Supplementary Figure S5). Our current results are in agreement with a previous in silico analysis of heparanase crystal structure and potential hot spots for small molecule drug design (unpublished studies performed by Dr. Fabian Glaser; The Lorry Lokey Interdisciplinary Center for Life Sciences and Engineering, Technion). It was found that while the enzymatic active site is a wrong site for drug design, there is a highly favorable docking site located very close and touching HBD-II, predicting that ligand binding there should block the substrate binding. Notably, the binding epitope for A54 mAb is retained in the proenzyme, rationalizing the ability of the antibody to bind to pro-heparanase and inhibit its cellular uptake and maturation. Collectively, we have generated and characterized a mAb that specifically neutralizes the heparanase enzyme and attenuates its pro-tumorigenic effects in preclinical models, paving the way for its clinical examination against cancer, inflammation, and other diseases.

## 5. Conclusions

We have generated and characterized a novel monoclonal antibody (A54 mAb) that specifically neutralizes heparanase enzymatic activity and attenuates its pro-tumorigenic effects. Mechanistically, co-crystallization of the A54 mAb and the heparanase enzyme indicated that the interaction between the two proteins takes place adjacent to the enzyme’s HS/heparin-binding domain II (HBDII), thereby hindering heparanase from interacting with HS substrates via steric occlusion of the active site cleft. A54 mAb attenuates xenograft tumor growth and metastasis primarily when administered in combination with conventional anti-cancer drugs, paving the way for its clinical examination against cancer, inflammation, and other diseases.

## Data Availability

The original contributions presented in this study are included in the article. Further inquiries can be directed to the corresponding authors.

## Supplementary Materials

The following supporting information can be downloaded at https://www.mdpi.com/article/10.3390/cells14171379/s1; Figure S1: A54 attenuates EMT-6 mouse breast carcinoma spontaneous metastasis; Figure S2: Humanized A54 mAb inhibits heparanase enzymatic activity; Figure S3: Humanized A54 mAb attenuates Panc02 mouse PDAC; Figure S4: Reciprocal space plots from STARANISO analysis of diffraction data; Figure S5: Overlay of heparanase catalytic residues between the structure of the A54 complex and the free protein; Table S1: Crystal structure data collection and refinement statistics; Table S2: PISA analysis of interactions between A54 VH chain and heparanase; Table S3: PISA analysis of interactions between A54 VL chain and heparanase.

## Abbreviations

The following abbreviations are used in this manuscript:

ECM	Extra Cellular Matrix
FCS	Fetal Calf Serum
kDa	Kilodalton
HS	Heparan Sulfate
Hpa	Heparanase
IgG	Immunoglobulin
IVIS	In Vivo Imaging System
HBD	Heparin Binding Domain
mAb	Monoclonal Antibody
PDB	Protein Data Bank
PBS	Phosphate-Buffered Saline
PDAC	Pancreatic Ductal Adenocarcinoma
PF4	Platelet Factor 4
SEM	Standard Error of the Mean
