# Supplementary material for: Heparanase-Neutralizing Monoclonal Antibody (mAb A54) Attenuates Tumor Growth and Metastasis
Source: Cells. 2025 Sep 4;14(17):1379. doi: 10.3390/cells14171379 (PMC12428753; doi:10.3390/cells14171379)
Supplement: Supplementary file 1 [file cells-14-01379-s001.zip › cells-3785596-supplementary.pdf]

Supplementary Materials

Article: **Heparanase-neutralizing monoclonal antibodies (mAb) attenuate tumor progression**

Uri Barash, Malik Farhoud, Maali Odeh, Eliezer Huberman, Liang Wu, and Israel Vlodavsky

Materials & Methods

Heparanase enzymatic activity - ECM degradation assay.

The extracellular matrix (ECM) substrate is deposited by cultured endothelial cells and hence closely resembles the subendothelial basement membrane in its composition, biological function and barrier properties (31). Briefly, sulfate [^{35}S] labeled ECM, prepared as described [31] and coating the surface of 35 mm tissue culture dishes, is incubated (4 h, 37 °C, pH 5.8, 1 ml final volume) with recombinant human heparanase (200 ng/ml) in the absence and presence of the A54 mAb. The reaction mixture contains: 50 mM NaCl, 1 mM DTT, 1 mM $CaCl_2$, and 10 mM buffer Phosphate-Citrate, pH 5.8 [30, 32]. To evaluate the release of proteoglycan/HS degradation fragments, the incubation medium is collected and applied for gel filtration onto Sepharose 6B columns (0.9 x 30 cm). Fractions (0.2 ml) are eluted with PBS and counted for radioactivity. The excluded volume (Vo) is marked by blue dextran, and the total included volume (Vt) by phenol red. Degradation fragments of HS side chains are eluted from Sepharose 6B at $0.5 < Kav < 0.8$ (peak II). Results are best represented by the actual gel filtration pattern [30, 32]. For inhibition studies, recombinant heparanase (200 ng) was first incubated with the indicated hybridoma or antibody for 30 min on ice at pH 7.2 and then incubated at pH 5.8 with the ^{35}S-labelled ECM, used as a substrate for the heparanase enzyme [31, 32].

Matrigel invasion assay.

Invasion assay was performed using modified Boyden chambers with polycarbonate Nucleopore membrane, essentially as described [36, 37]. Briefly, filters (6.5 mm in diameter, 8 µm pore-size) were coated with Matrigel (30 µl); Cells (2 x 10^5), suspended in 100 µl of serum-free medium, were seeded in triplicate on the upper part of each chamber in the absence or presence of the A54 mAb, and the lower compartment was filled with 600 µl medium supplemented with 10% FCS. After incubation for 6 h at 37°C in a 5% CO_2 incubator, non-invading cells on the upper surface of the filter were wiped with a cotton swab, and invasive cells on the lower surface of the filter were fixed, stained with 0.5% crystal violet (Sigma) and counted by examination of at least seven microscopic fields [36].

U87 glioma.

Luciferase-labeled U87 glioma cells were detached with trypsin/EDTA, washed with PBS, and brought to a concentration of $5x10^7$ cells/ml. Cell suspension ($5x10^6$/0.1 ml) was inoculated subcutaneously (s.c.) at the right flank of 5-week-old female NOD/SCID mice. Three days after cell inoculation, mice are randomly assigned to two groups (n = 5 mice each) receiving vehicle (PBS) or A54 mAb (500 µg/mouse, i.p., 3 times/week). Tumor development was inspected (once a week) by IVIS imaging, following administration of luciferin (see below) as described [30, 34]. At the end of the experiment, mice are sacrificed, and xenografts are resmoved, weighed, and fixed in formalin for pathological examination.

IVIS imaging.

Bioluminescent imaging of luciferase-expressing tumors is performed with a highly sensitive, cooled charge-coupled device (CCD) camera mounted in a light-tight specimen box (IVIS; Xenogen Corp., Waltham, MA). Imaging is performed in real time, is non-invasive and provides quantitative data. Briefly, mice are injected intraperitoneally with D-luciferin substrate at 150 mg/kg, anesthetized and placed onto a warmed stage inside the light-tight camera box, with continuous exposure to isoflurane (EZAnesthesia, Palmer, PA). Light emitted from the bioluminescent cells is detected by the IVIS camera system, as described [30].

*The indicated references are the same as those referred to in the main article file.

Figures and Tables

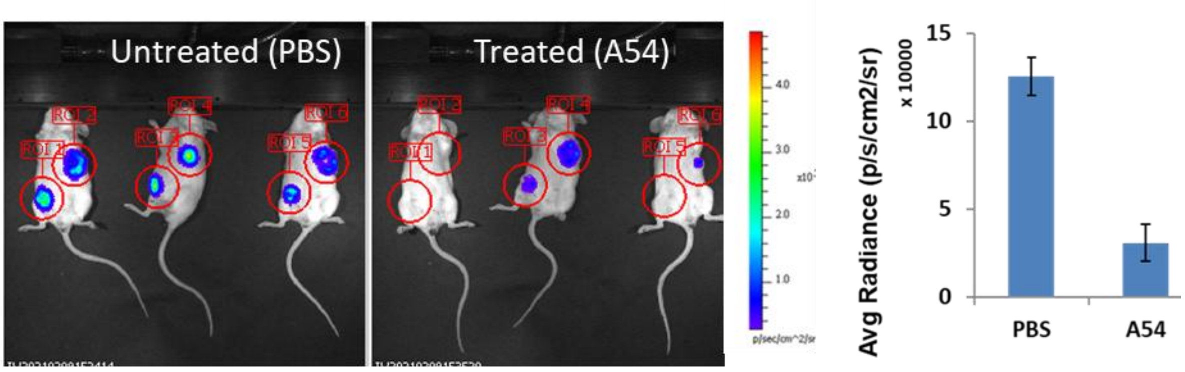

Supp Fig. S1. A54 attenuates EMT-6 mouse breast carcinoma spontaneous metastasis. Luciferase-labeled EMT-6 breast carcinoma cells ($1x10^5$/Balb/c mouse) were injected into the third mammary fat pad, and treatment of mice (n=3) with PBS or mAb A54 (360 µg/mouse, 3 times/week) began 3 days after EMT-6 cell inoculation. On day 15 of the study, the mammary fat pad, including the primary tumor, was excised, and the mice were further treated with mAb A54 as described above. IVIS bioluminescent imaging was performed 20 days after mastectomy.

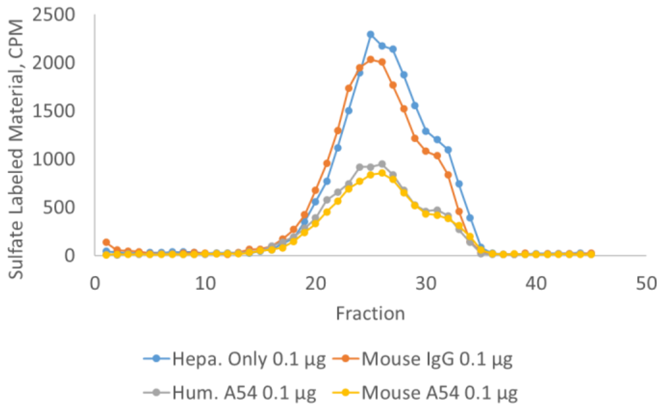

Supp Fig. S2. Humanized A54 mAb inhibits heparanase enzymatic activity. Purified recombinant active heparanase (200 ng) was pre-incubated alone (blue), or with control mouse IgG (orange), mouse A54 (yellow), or humanized A54 mAb (gray) at 1 µg/ml for 1 h in serum-free RPMI medium on ice. The mixture was then incubated (3h, 37 °C) with 35S-labeled ECM, and heparanase enzymatic activity (release of sulfate-labeled fragments of HS) was determined as described in 'Materials and Methods'.

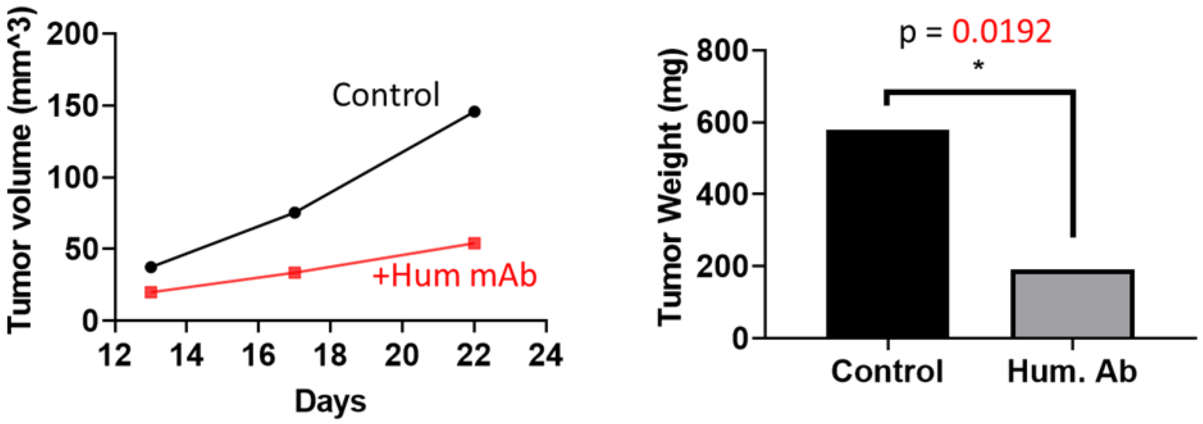

Supp Fig. S3. Humanized A54 mAb attenuates Panc02 mouse PDAC. C57BL/6 mice (n=5) were inoculated (s.c.) with Panc02 cells (1×10^{6}/mouse/0.1 ml). Mice were treated with humanized A54 mAb (i.p. 640 μg/mouse, 3 times/week). Tumor volume was measured on days 13, 17, and 22 (left). At the end of the experiment on day 26, tumors were resected and weighed (right).

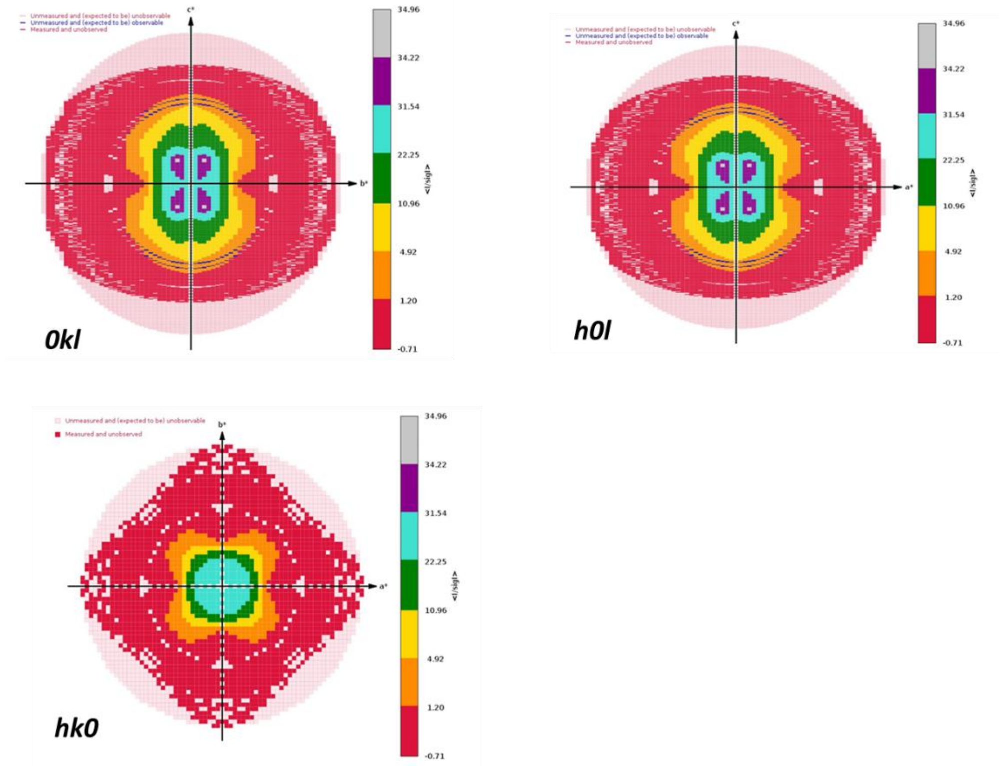

Suppl Figure S4: Reciprocal space plots from STARANISO analysis of diffraction data (ref 39**).**

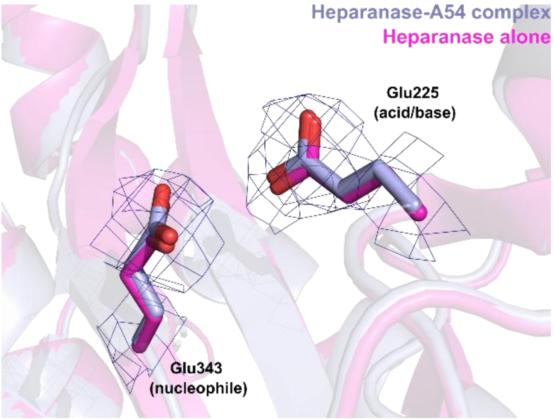

Suppl Figure S5. Overlay of heparanase catalytic residues Glu343 (nucleophile) and Glu225 (acid/base) between the structure of the A54 complex (blue), **and the free protein** (PDB 5E8M; pink). A54 binding does not perturb the positions of the catalytic sidechains within the heparanase active site. Sidechain electron density, corresponding to the A54 complex, is REFMAC σ_A-weighted 2mFo–DFc, contoured to 1.0 σ (0.28 e^{-}.Å^{-3}).

Supp Table 1 - Crystal structure data collection and refinement statistics

	A54-heparanase complex
**Data collection**	
Space group	P41 21 2
Cell dimensions	
a, *b*, *c* (Å)	69.74, 69.74, 429.78
α, β, γ (°)	90, 90, 90
Resolution (Å)	107.44-3.49 (3.87-3.49)
R_{meas}	0.28 (2.25)
$I / \sigma I$	9.2 (1.7)
Completeness (ellipsoidal; %)	86.8 (59.0)
Redundancy	15 (16.7)
**Refinement**	
Resolution (Å)	107.44-3.49
No. reflections	9227
R_{work} / R_{free}	0.20/0.32
No. atoms	
Protein	6987
Ligand/ion	66
Water	29
B-factors	
Protein	145.5
Ligand/ion	159.8
Water	61.2
R.m.s. deviations	
Bond lengths (Å)	0.014
Bond angles (°)	2.05

Values in parentheses are for the highest resolution shell.

Diffraction limits (mean I/σI > 1.2) & principal axes of ellipsoid fitted to diffraction cut-off surface:

4.190	1.0000	0.0000	0.0000	_a_*
4.190	0.0000	1.0000	0.0000	_b_*
3.367	0.0000	0.0000	1.0000	_c_*

Supp Table 2 – PISA analysis of interactions between A54 V_H chain and heparanase

Interfacing residues - A54 V_H chain

Amino acid	*Residue number*	*H-bond/Salt bridge?*	*ASA*	*BSA*	*DeltaG*
H:TYR	69		34.85	1.6	-0.02
H:ILE	70		10.7	3.18	0.05
H:THR	73		52.25	34.8	0.2
H:THR	74	H	63.46	62.96	0.27
H:GLY	75	H	48.27	44.65	0.13
H:TYR	76	H	76.32	76.05	0.22
H:THR	77	H	49.9	10.14	-0.05
H:GLU	78	S	81.71	52.19	-0.4
H:TYR	79		47.39	1.6	-0.02
H:GLN	81		140.64	27.9	-0.29
H:LYS	84		110.27	43.71	-0.47
H:ALA	91		35.7	0.29	0
H:LYS	93	HS	125.29	58.57	-1.07
H:TYR	122		110.63	70.64	0.7
H:ASP	123	S	65.43	59.03	-0.27
H:TYR	124		83.85	65.22	0.75
H:ASP	125	HS	137.18	100.86	-0.28
H:GLU	126		71.9	18.64	-0.26
H:ASP	127	H	20.18	10.12	-0.12
H:TYR	128		189.39	8.87	0.14

Interfacing residues - heparanase

Amino acid	*Residue number*	*H-bond/Salt bridge?*	*ASA*	*BSA*	*DeltaG*
A:LEU	230		84.91	25.55	0.41
A:LYS	231	S	126.88	59.98	0.43
A:GLN	270	H	56.36	32.2	-0.22
A:PRO	271		20.13	12.86	-0.09
A:ARG	272		110.41	76.22	0.79
A:ARG	273	HS	197.53	75.86	-0.83
A:ALA	276		21.85	1.51	0.02
A:TYR	298	H	15.64	0.49	-0.01
A:TYR	299	H	15.55	15.55	-0.17
A:LEU	300		28.93	28.6	0.46
A:ASN	301	H	41.87	27.94	-0.04
A:THR	304	H	97.93	25.48	0.08
A:ALA	305		16.47	1.5	0.02
A:THR	306	H	53.39	19.25	0.31
A:GLU	308		113.29	10.65	-0.12

A:ASP	309	S	38.28	34	-0.41
A:ASN	312		50.04	9.66	-0.11
A:ASP	314		77.37	29.64	0.17
A:VAL	315		22.8	21.21	0.34
A:ILE	318		77.71	61.32	0.95
A:SER	321		45.1	13.33	-0.12
A:SER	322		13.91	12.24	-0.11
A:LYS	325	S	97.11	52.82	-1.15
A:TYR	348	H	101.7	46.39	-0.04

Supp Table 3 – PISA analysis of interactions between A54 V_L chain and heparanase

Interfacing residues - A54 V_L chain

Amino acid	Residue number	H-bond/Salt bridge?	ASA	BSA	DeltaG
L:ASP	20		109.76	15.25	-0.14
L:ILE	21		0.17	0.17	0
L:GLU	46		117.79	5.24	-0.06
L:SER	47		46.58	0.37	0
L:GLU	49		71.2	1.59	-0.02
L:TYR	50		157.02	100.71	0.69
L:PHE	51		201.18	6.25	0.1
L:TYR	55		65.82	25.87	0.21
L:SER	114	H	27.53	10.36	-0.12
L:ASN	115		36.95	35.62	-0.33
L:GLU	116	S	69.24	51.05	0.25
L:ASP	117	S	125.73	67.54	-0.76
L:PRO	118		71.47	2.34	0.04
L:TYR	119		121.81	4.68	-0.03

Interfacing residues - HPSE

Amino acid	Residue number	H-bond/Salt bridge?	ASA	BSA	DeltaG
A:LEU	230		84.91	14.9	0.24
A:LYS	231		126.88	4.69	0.07
A:ARG	272	S	110.41	25.87	-0.09
A:ARG	273	H	197.53	121.67	-0.43
A:LYS	274		71.84	49.52	0.51
A:ALA	276		21.85	16.28	0.19
A:LYS	277	S	142.35	85.08	-1.16
A:LYS	280		75.86	1.51	0.02
A:GLN	328		121.39	5.98	0.05
A:VAL	329		19.74	10.21	0.16
